# Effect of antiretroviral therapy on fertility rate among women living with HIV in Tabora, Tanzania: An historical cohort study

**DOI:** 10.1371/journal.pone.0222173

**Published:** 2019-09-06

**Authors:** Gaspar Mbita, Jenny Renju, Gissenge Lija, Donaldson F. Conserve, Jim Todd

**Affiliations:** 1 Kilimanjaro Christian Medical University College (KCMCUCo), Moshi, Tanzania; 2 Jhpiego Tanzania, Dar es Salaam, Tanzania; 3 Department of Population Health, London School of Hygiene and Tropical Medicine, London, United Kingdom; 4 National AIDS Control Program, Ministry of Health, Community Development, Gender, Elderly and Children, Dodoma, Tanzania; 5 Department of Health Promotion, Education, and Behavior, Arnold School of Public Health, University of South Carolina, Columbia, South Carolina, United States of America; University of the Witwatersrand, SOUTH AFRICA

## Abstract

The modelling of HIV trends in Tanzania uses surveillance data from antenatal clinics after adjusting for the reduction in fertility of women living with HIV (WLWH). The rollout of HIV care and treatment services has enabled many WLWH to start on antiretroviral treatment (ART) earlier and are counselled on the options to prevent HIV transmission to their children. The assumption that being HIV positive leads to lower fertility needs to be revisited. This study aims to quantify the effect of ART program expansion on the fertility rate of WLWH in Tanzania. We used Cox regression model to estimate fertility rate and associated factors among WLWH of reproductive age (15–49 years) who enrolled in HIV care and treatment at 57 centers in Tabora from 2008 to 2014. A decomposition of Poisson regression was used to explore the reasons for fertility rate differences observed among WLWH. A total of 6,397 WLWH aged 15–49 years were followed for a median time of 2.0 years. The total fertility rate of 48.8/1,000 person years (95%CI: 44.6 to 52.9/1,000) was inversely proportional to age and WHO clinical staging. WLWH on ART had higher fertility compared to those not started on ART (aHR = 1.5, 95%CI: 1.2–1.9). Being married or cohabiting, having higher CD4 cell count and not using contraceptives were associated with higher fertility rate. The fertility rate after post-ART initiation was 54.95/1,000 and among pre-ART users was 40.52/1,000, a difference of 14.43/1,000 in fertility rate between the groups. In the decomposition analysis, proximate determinants of fertility rate among WLWH on ART accounted for a 93.8% smaller increase than expected. In an era of ART expansion in Tabora region, fertility rates of WLWH increased. Higher fertility rates in women on ART may alter the estimation of HIV prevalence and incidence.

## Introduction

The HIV epidemic continues to be a major public health problem around the world. In 2017, approximately 37 million people were living with HIV (PLWH), of whom 25 million are in sub-Saharan Africa [1]. The 2017 Tanzania Health Impact Survey (THIS) reported approximately 1.4 million PLWH in Tanzania, a prevalence of 5.0% among adults aged 15 to 64 years (6.5% among females and 3.5% among males) [2].

Early in the pandemic, the relationship between HIV/AIDS and fertility was determined to be largely biological and behavioral [3]. Some studies in sub-Saharan Africa reported that prior to widespread availability of antiretroviral therapy (ART) there was an inverse relationship between HIV/AIDS and fertility, particularly during the advanced stages of infection [4]. Fertility was lower among women living HIV (WLWH) than those living without HIV, with the exception of those aged 15–19 years (3–5).

The Total Fertility Rate (TFR) in Tanzania was 5.7 births per woman in 2005, 5.4 in 2010 and 5.2 in 2015 [5]. Routinely collected data from antenatal clinics (ANC) are used to generate country-specific trends in HIV prevalence to monitor the effectiveness of prevention and control measures, and are still using the assumptions of reduced fertility of WLWH in making these estimates [6].

The rapid expansion of HIV Care and Treatment services has increased the number of people taking ART with 21.1 million people on ART worldwide, and of these, 12.9 million are in Eastern and Southern Africa [1]. In Tanzania, nearly all (91%) PLHIV aged 15 to 64 years who knew their HIV status were receiving ART in 2016 [2]. The rollout has contributed to a greater awareness of issues related to fertility and childbearing among WLWH [7]. Some studies have reported an increasing overall fertility rate among WLWH and a smaller difference in fertility rates between WLWH and those living without HIV [8]. Therefore, the increased access to care and treatment services and ART uptake may affect the fertility of WLWH implying a need to re-estimate the adjustment factors used in calculations of country-specific HIV estimates.

In Tanzania, to date, only one study has examined the impact of ART on fertility and it focused on Kisesa in northwest Tanzania, and used population data from demographic surveillance [8]. The study reported that the difference in fertility between WLWH and those living without HIV are narrowing over time as ART becomes more widely available in communities but did not explain how much of the change is attributable to other factors than ART [8].

Our study contributes to the growing literature on ART and fertility in Sub-Saharan Africa. Routinely collected data from HIV Care and Treatment Centers (CTCs) in Tanzania was used to assess the impact of ART on the fertility rate among WLWH from 2008 to 2014 after observing several changes in guidelines for management of HIV and AIDS [9–13].

Additionally, many studies conducted in Sub-Saharan Africa have highlighted factors associated with fertility rates among people using ART and those who are not using ART medication. These factors include CD4 count, age, marital status, contraceptive use, body mass index (BMI) and World Health Organization (WHO) clinical staging [8, 14, 15]. The contribution of these factors on fertility rate change can be partitioned into two parts, the part that can be explained due to changes in the characteristics of the participants (endowments, explained variation) and the part that is due to changes in their coefficients in the statistical model (effect of the factors, unexplained variation). Most studies of factors associated with ART on fertility rate did not examine how these factors contribute (endowments and coefficients contributions) to the fertility rate changes among WLWH after initiation of ART. This study aimed at assessing the impact of ART on fertility and other factors that were associated with fertility rate among WLWH and estimates the contribution of each of these factors on fertility rate [16].

## Methods

### Clinical setting and data collection

The data was collected in Tabora region located in the central-western part of the country and one of Tanzania's 31 administrative regions. The region has seven councils, which are Tabora Municipal, Igunga, Kaliua, Nzega, Sikonge, Urambo and Uyui district councils. The large part of the area is semi-urban. Agriculture providing the main source of income and the majority of people living in Tabora are Sukuma and Nyamwezi. According to the 2012 population and housing census, there 2,291, 623 people living in Tabora with an average household size of 6.0 and sex ratio of 97 females to 100 males [17].

This study uses retrospective cohort analysis of de-identified routinely collected data from the national HIV patient electronic database at 57 HIV clinics in Tabora, Tanzania. Patients from these facilities were receiving HIV services on a regular basis and their demographic characteristics, clinical examinations, treatment outcomes and pregnancy status were captured in paper records and later entered into on-site electronic databases. The National AIDS Control Program (NACP) performed initial data cleaning. De-identified datasets were exported by NACP and provided to the investigators for analysis.

Medical officers and medical assistants mainly provided HIV care and treatment for pregnant women, who then received ART from pharmacy assistants or nurses within the prevention of mother-to-child transmission (PMTCT) section of ANC. HIV care and treatment services, including CD4 cell count testing and ART, were provided at no charge to clients at all government clinics.

At the first clinic visit (enrolment visit), demographic characteristics including age in years, gender and marital status were captured. A clinical assessment including weight in kilograms, height in centimeters, WHO clinical staging and CD4 cell count were performed and information on family planning use was collected. Monthly follow-up visits were recommended for patients followed before (pre- ART) and after ART initiation (post-ART initiation). At follow-up visits, the clinical and ART status of each patient was reviewed and documented. Appropriate clinical management (including ART initiation for those eligible) was provided as per national guidelines. At this time, guidelines stated that CD4 cell count testing to assess ART eligibility should be repeated every six months or more frequently if needed [13]. Health facilities without a CD4 cell count machine out-sourced testing and the return of results depended on the distance and availability of transport to and from the testing facility.

### Data management and analysis

This analysis includes data of WLWH aged 15–49 years at 57 clinics in Tabora region enrolled from January 2008 to December 2014. During this time, ART was initiated according to the 2008 WHO recommendations/2009 Tanzania guidelines, which recommended ART initiation for all patients with WHO Stage 3 or 4 irrespective of CD4 cell count or patients with CD4 cell count below 350cells/mL irrespective of the WHO stage [10]. Women with ART enrollment information only and no follow-up information were excluded from the analysis. Women who reported having received sterilization as a family planning method were excluded at the date of reported sterilization. The dataset was closed on 31 June 2015, after each woman had at least six months of follow-up visits.

Missing information on age, weight, length, CD4 cell count, and WHO clinical staging at first visit were imputed from the closest measurements in the next visit of the same woman. The main outcome of interest was new pregnancies and a woman may have multiple pregnancies during the follow-up time. Women who became pregnant during follow-up were censored at their first visit in which they reported the pregnancy and uncensored twelve months after delivery of the pregnancy. Fertility rates were calculated over the person-years from the date of first clinic visit to the study until the date of censoring. Women were censored when they became pregnant, died, or reached their 50^th^ birthday. The follow up time was censored 90 days after the last clinic visit (last follow-up) or on 30 June 2015 (the end of the study). Women newly diagnosed with HIV during their pregnancy were excluded from the analysis until 12 months after delivery. For women who had undergone sterilization during the follow-up period were censored from the date of sterilization. The follow-up period of women prior to initiation of ART was designated as the ‘‘pre-ART” period, while follow-up period after initiation of ART was designated as the ‘‘post-ART initiation”. The fertility rates were calculated per 1,000 person years. The fertility rate ratio from Mantel-Haenszel test with 95% confidence intervals was used to compare the fertility rates across all levels of explanatory variables. The Cox regression model was used to assess the factors that were independently associated with fertility rate. The robust variance was used to control for health facility variability.

Multivariate decomposition models were used to assess association of various participant characteristics on the changes in fertility rate among post-ART initiators. Generally, the model partitions changes of fertility rate into two parts, namely, the part that is due to changes in characteristics of the variables (endowments, explained variation) and the part that is due to changes of the coefficients of these variables for post-ART initiators (effect of the factors themselves). All analyses were conducted in Stata 13.0 (StataCorp, College Station, TX).

## Ethical considerations

Ethical approval was received from the Kilimanjaro Christian Medical University College Research and Ethics Review Committee No. 886, 2015. A Data Transfer Agreement from National AIDS Control Program (NACP) was obtained in order to use the patient’s level information from the CTC. Personal identifications including names, phone numbers, and household identifiers were removed from the database by NACP.

## Results

### Baseline characteristics of participants

**Fig 1:**
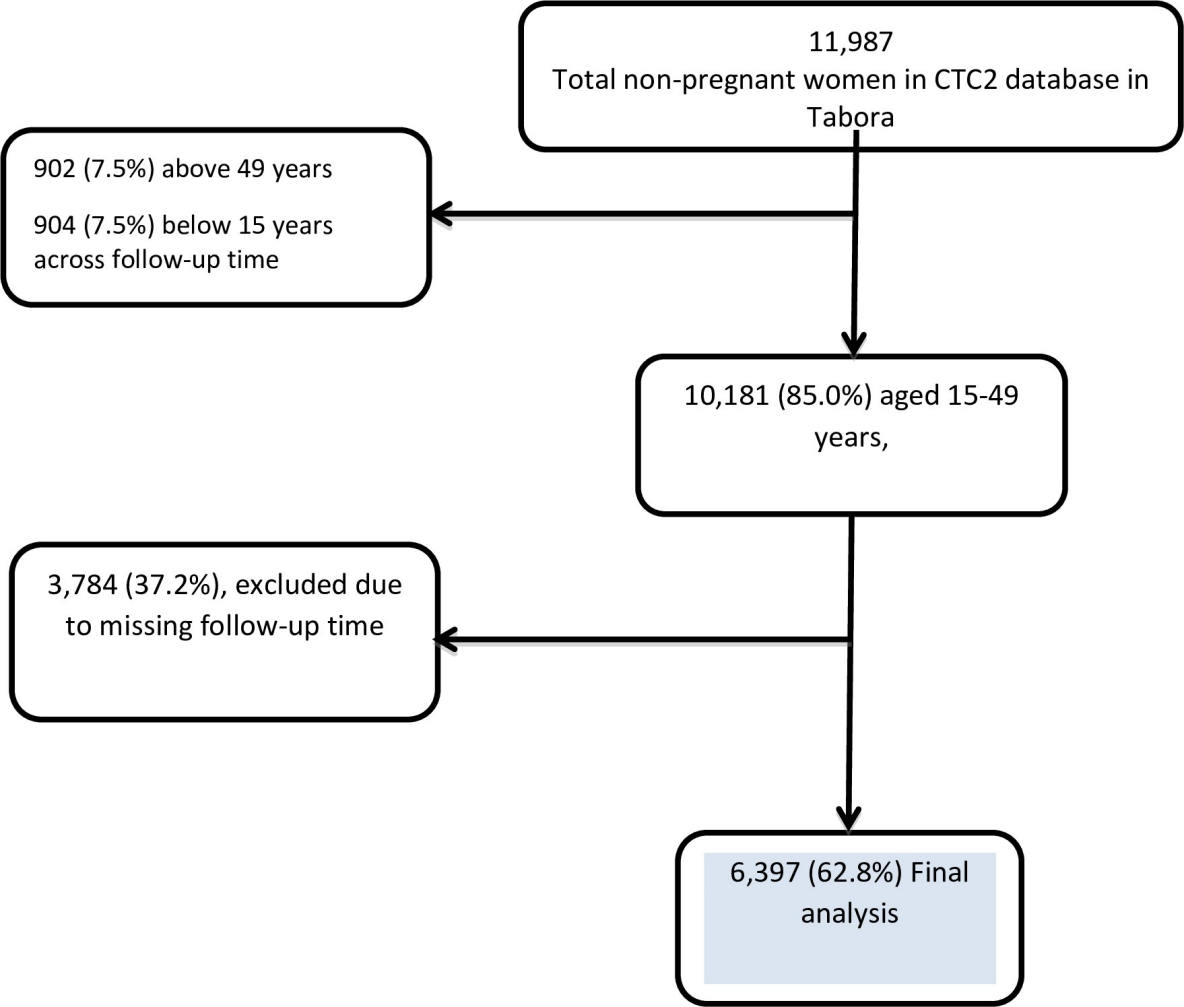
The flow chart of women enrolled at the clinic and those included in the final analysis.

Information of 11,987 non-pregnant WLWH were extracted from the NACP database, with 10,181 (85%) aged 15 to 49 years at the time of enrolment. A total of 3,784 (37.2%) women were excluded from the analysis as only one visit was recorded in the NACP database, leaving 6,397 women included for the analysis (Fig 1).

Comparison of those who were included in the study (6,397) and the participants excluded from the final analysis (3,784) indicated that there were no significant differences with regard to age, marital status and contraceptive use (data not shown). Majority of those excluded (82%) were not on ART.

The maximum study follow-up time was 7.4 years, with a median follow-up time of 2.0 years.

**Fig 2:**
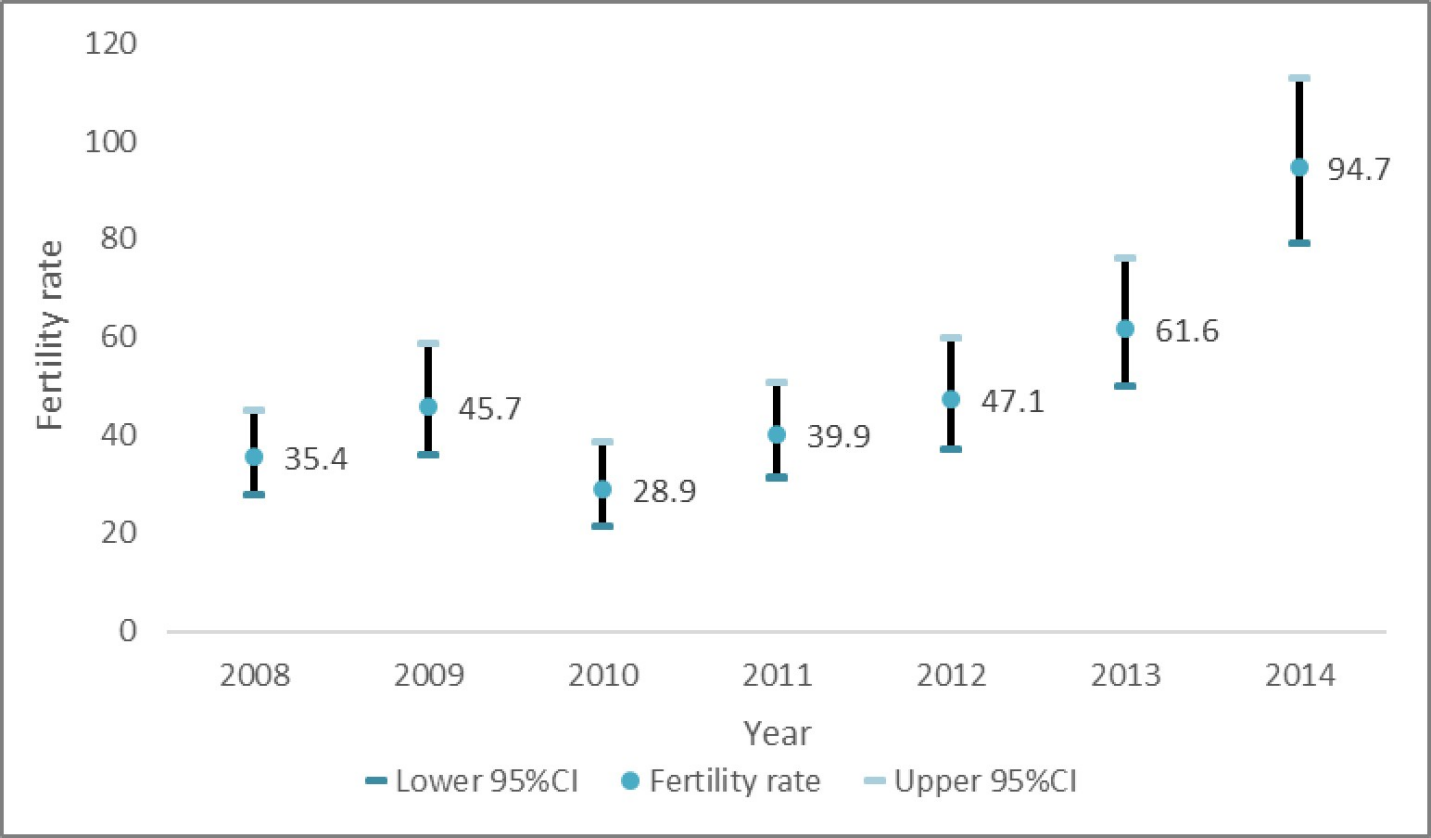
Trend in fertility rates in women living with HIV from 2008 to 2014.

A cohort 6,397 WLWH from 2008 to 2014 was followed for a total of 10,800 years (median 2.0 years). We observed 523 new pregnancies. The total fertility rate was 48.5/1,000, person years. The age-specific fertility rates were higher among younger women (96.1/1,000 in ages 20–24 and 91.5/1,000 in ages 15–19 years), and decreased with increasing age. Women who were married or cohabiting had higher fertility (56/1,000) compared to those who were single (36.5/1,000) or divorced/widowed (37.2/1,000). Women who were not using any contraception method had higher fertility rate (57.0/1,000) than those reported using contraception (6.6/1,000). Furthermore, we observed an upward trend in fertility over time from the year 2008 to 2014. The fertility was 35.4/1,000 person years in 2008 and this fertility rate was almost three times higher six years later (94.7/1,000 years in 2014). In 2010, the fertility rate dropped from 45.7/1,000 person years in 2009 to 28.9/1,000 in 2010 (Table 1 and Fig 2).

**Table 1:** Baseline characteristics of women living with HIV included in the final analysis.

Factors	Total women (%)	Person years (in 1000)	Pregnancies (%)	Pregnancy rate/1000 person years	95% CI/1000 person years
**All patients**	**6,397 (100)**	**10.8**	**523**	**48.5**	**44.6–52.9**
**Age (Years)**					
15–19	783 (12.2)	1.0	89	91.5	74.3–112.6
20–24	1,262 (19.7)	1.8	169	96.1	82.7–111.8
25–29	1,322 (20.7)	2.2	145	64.8	55.1–76.2
30–34	1,272 (19.9)	2.4	84	34.6	28.0–42.9
35–39	807 (12.6)	1.5	24	15.6	10.4–23.3
40–49	951 (14.9)	1.8	12	6.5	3.7–11.5
**Marital status**					
Single	1,260 (19.7)	1.9	71	36.5	28.9–46.1
Divorced/Widowed	1,392 (21.8)	2.3	84	37.2	30.0–46.0
Married/Cohabiting	3,745 (58.5)	6.6	368	56.0	50.5–62.0
**CD4 cell count**					
**≤** 350	2,739 (42.8)	4.1	189	46.2	40.1–53.3
**>** 350	3,658 (57.2)	6.7	334	49.9	44.9–55.6
**Contraceptive use**					
Using	1,821 (28.5)	1.8	12	6.6	3.8–11.6
Not using	4,576 (71.5)	9.0	511	57.0	52.3–62.2
**BMI**					
Above 24Kg/m^2^	508 (8.0)	1.1	54	50.0	38.3–65.2
18-24Kg/m^2^	3,201 (50.0)	6.2	329	53.2	47.7–59.2
Below 18Kg/m^2^	2,688 (42.0)	3.5	140	39.9	33.8–47.1
**WHO Clinical staging**					
WHO stage IV	888 (13.9)	1.8	62	34.3	26.8–44.1
WHO stage III	2,050 (32.1)	3.6	133	37.1	31.3–43.9
WHO stage II	1,882 (29.4)	2.9	132	45.8	38.6–54.3
WHO stage I	1,577 (24.7)	2.5	196	78.5	68.2–90.3
**Year**					
2008	1,066 (16.7)	2.0	69	35.4	27.9–44.8
2009	758 (11.9)	1.4	63	45.7	35.7–58.5
2010	854 (13.4)	1.6	46	28.9	21.6–38.6
2011	909 (14.2)	1.7	69	39.9	31.5–50.5
2012	891 (13.9)	1.4	68	47.1	37.1–59.7
2013	924 (14.4)	1.4	85	61.6	49.8–76.2
2014	995 (15.6)	1.3	123	94.7	79.3–113.0

### Fertility rates pre- and post- ART initiation

In pre-ART, we observed 3,413 (53.4%) women and 2,984 (46.6%) post-ART initiation. The total fertility in the post-ART group was 55.0/1000 compared to 40.5/1000 in pre-ART group giving an overall fertility rate 1.3 times higher in women on ART compared to those who are not on ART (RR = 1.3, 95% CI 1.1–1.6) (Table 2).

**Table 2:** Total fertility of participants by pre-ART and post-ART status.

Factor	Total women	Pre-ART			Post-ART		
		Person-years	Pregnancy	fertility Rate/1000 Rate(95%CI)	Person-years	Pregnancy	fertility Rate/1000 Rate(95%CI)
**All women**	**6,397**	**4.8**	**194**	**40.5(35.2–46.7)**	**6.0**	**329**	**55.0(49.3–61.2)**
**Age (Years)**							
15–19	783	0.5	37	70.9(51.4–97.9)	0.5	52	115.2(87.8–151.2)
20–24	1,262	0.8	68	81.1(64.0–102.9)	0.9	101	109.8(90.3–133.4)
25–29	1,322	0.9	47	49.7(37.4–66.2)	1.3	98	75.8(62.2–92.4)
30–34	1,272	1.0	29	28.2(19.6–40.5)	1.4	55	39.4(30.2–51.3)
35–39	807	0.7	10	15.2(8.2–28.4)	0.9	14	15.9(9.4–26.8)
40–49	951	0.8	3	3.8(1.2–11.6)	1.1	9	8.6(4.5–16.5)
**Marital status**							
Single	1,260	0.8	21	25.8(16.8–39.6)	1.1	50	44.2(33.5–58.3)
Divorced/Widowed	1,392	1.0	24	25.0(16.7–37.3)	1.3	60	46.2(35.9–59.5)
Married/Cohabiting	3,745	3.0	149	49.4(42.1–58.1)	3.6	219	61.6(53.9–70.3)
**CD4 cell count**							
**≤** 350	2,739	1.8	57	32.0(24.7–41.5)	2.3	132	57.2(48.2–67.8)
**>** 350	3,658	3.0	137	45.5(38.5–53.8)	3.7	197	53.6(46.6–61.6)
**Contraceptive**							
Using	1,821	0.7	1	1.5(0.2–10.6)	1.1	11	9.6(5.3–17.4)
Not using	4,576	4.1	193	46.9(40.7–54.0)	4.8	318	65.6(58.8–73.3)
**BMI**							
Above 24Kg/m^2^	508	0.4	15	35.7(21.5–59.2)	0.7	39	59.1(43.2–80.9)
18-24Kg/m^2^	3,201	2.6	114	43.6(36.3–52.4)	3.6	215	60.1(52.6–68.7)
Below 18Kg/m^2^	2,688	1.8	65	37.0(29.0–47.2)	1.8	75	42.8(34.1–53.7)
**WHO Staging**							
WHO stage IV	888	0.5	16	29.4(18.0–48.1)	1.3	46	36.5(27.3–48.7)
WHO stage III	2,050	1.3	25	18.7(12.6–27.7)	2.3	108	48.0(39.7–57.9)
WHO stage II	1,882	1.5	63	42.6(33.3–54.6)	1.4	69	49.1(38.8–62.1)
WHO stage I	1,577	1.4	90	62.9(51.1–77.4)	1.1	106	99.3(82.1–120.2)
**Year**							
2008	1,066	1.3	41	32.0(23.6–43.5)	0.7	28	41.7(28.8–60.4)
2009	758	0.8	32	40.9(28.9–57.8)	0.6	31	52.0(36.6–74.0)
2010	854	0.7	20	29.7(19.1–46.0)	0.9	26	28.3(19.3–41.6)
2011	909	0.8	26	33.4(22.8–49.1)	1.0	43	45.1(33.5–60.9)
2012	891	0.6	28	47.4(32.7–68.6)	0.9	40	46.9(34.4–64.0)
2013	891	0.4	30	71.1(49.7–101.7)	1.0	55	57.4(44.1–74.7)
2014	995	0.3	17	65.4(40.7–105.2)	1.0	106	102.0(84.3–123.4)

Women aged 15–24 years old had a higher fertility rate (for pre-ART: aged 15–19 years was 70.9/1,000 and 20–24 was 81.1/1,000 and for post-ART: aged 15–19 years was 115.2/1,000 and 20–25 was 109.8/1,000) compared to women of older ages for both pre-ART and post-ART groups. Little difference was noted between the pre and post ART groups amongst older women (over 39 years) for example women between 35–39 years had a pre-ART fertility rate of 15.2/1000 compared to a post-ART fertility rate of 15.9/1000. The fertility rates in post-ART and pre-ART also differed by marital status, whereby women who were in the post ART group and married or cohabiting had a higher fertility rate (61.6/1000 compared to 49.4/1000 in pre-ART group. We observed a small difference in fertility between pre-ART (28.3/1000, 95%CI 19.3–41.6/1000) and post-ART in 2010 (29.7/1000, 95%CI (19.1–46.0/1000) [9] and bigger difference in 2014, 102/1000 (95%CI 84.3–123.4) in post-ART group compared to 65.4/1000 (95%CI 40.7–105.2) in pre-ART group (Table 2).

### The effect of ART and other factors on fertility rate

We predicted the effect of ART on fertility by controlling for health facility variabilities, age, reported marital status, and reported modern family planning use. The fertility rate of women in the post-ART group was 50% higher than women in the pre-ART group (adjusted HR (AHR) = 1.5, 95%CI: 1.2–1.9). The fertility rate decreased with increasing age, where women aged 40–49 years had a 90% lower fertility rate than women aged between 15 to 19 years (AHR = 0.1, 95%CI: 0.03–0.1). The fertility rate of married or cohabiting women was 1.5 times higher than women who reported to be single (AHR = 1.5, 95%CI: 1.2–2.0). The fertility rate of women with a CD4 count of 350 cells or more was 20% higher than women with a CD4 count of less than 350 cells (AHR = 1.2, 95%CI: 1.0–1.3). The fertility rate of women with a lower WHO clinical staging (stage one) were two-fold higher than those classified at WHO stage four (AHR = 2.3, 95%CI: 1.7–3.1) and the fertility rate of the women enrolled in the study in the year 2014 was twice as high as the fertility rate of those who were enrolled in the year 2008. There was no statistical difference in the fertility rate of women who reported to have a BMI below 24kg/m^2^ compared to those with a BMI above 24kg/m^2^ (Table 3).

**Table 3:** Factors associated with fertility rate.

Factors	Crude HR (95% C.I.)	p-value (Likelihood ratio test)	adjusted HR (95% C.I.)	p-value (Likelihood ratio test)
**Age (Years)**				<0.0001
15–19	**1.0**		**1.0**	
20–24	1.0 (0.8–1.3)	0.753	1.0 (0.8–1.3)	
25–29	0.7 (0.5–0.9)	0.003	0.7 (0.5–0.9)	
30–34	0.3 (0.3–0.5)	<0.001	0.3 (0.2–0.5)	
35–39	0.2 (0.1–0.2)	<0.001	0.2 (0.1–0.3)	
40–49	0.1 (0.03–0.1)	<0.001	0.1 (0.03–0.1)	
**Marital status**				<0.0024
Single	**1.0**		**1.0**	
Divorced/Widowed	1.0 (0.7–1.4)	0.890	1.3 (0.9–1.9)	
Married/Cohabiting	1.5 (1.2–2.0)	0.001	1.5 (1.2–2.0)	
**ART status**				0.0001
Pre-ART	**1.0**		**1.0**	
On-ART	1.3 (1.1–1.6)	0.002	1.5 (1.2–1.9)	
**CD4 cell count**				0.0051
**≤** 350	**1.0**		**1.0**	
**>** 350	1.0 (0.9–1.2)	0.681	1.2 (1.0–1.3)	
**Contraceptive use**				<0.0001
Using modern family planning method	**1.0**		**1.0**	
Not using modern family planning method	8.7 (4.8–15.8)	<0.001	10 (5.7–21.0)	
**BMI**				0.1176
Above 24Kg/m^2^	**1.0**		**1.0**	
18-24Kg/m^2^	1.1 (0.8–1.4)	0.586	1.1 (0.8–1.4)	
Below 18Kg/m^2^	0.8 (0.6–1.1)	0.246	0.9 (0.7–1.1)	
**WHO Staging**				<0.0001
WHO stage IV	**1.0**		**1.0**	
WHO stage III	1.1 (0.8–1.6)	<0.001	1.2 (0.9–1.5)	
WHO stage II	1.5 (1.1–2.0)	<0.001	1.5 (1.1–2.0)	
WHO stage I	2.6 (1.9–3.4)	<0.001	2.3 (1.7–3.1)	
**Year of Enrollment**				<0.0001
2008	**1**			
2009	1.3 (0.9–1.9)	0.100	1.2 (0.7–2.0)	
2010	0.9 (0.6–1.2)	0.403	0.7 (0.5–0.9)	
2011	1.2 (0.8–1.6)	0.340	0.9 (0.6–1.3)	
2012	1.4 (1.0–2.0)	0.037	1.2 (0.7–2.2)	
2013	1.9 (1.4–2.6)	<0.001	1.5 (0.9–2.7)	
2014	2.7 (2.0–3.6)	<0.001	1.9 (1.1–3.2)	

### Decomposition analysis for factors associated with fertility rate

The fertility rate among post-ART was 54.95 births per 1000 person years and among pre-ART women was 40.52 births per 1000 person years, yielding an increased by 14.43 births per 1000 person years in women on ART, compared to those not on ART (Table 4). However, there was an increase in the number of women using contraceptives among women on ART (34.2%), compared to those not on ART (23.4%). If this had not happened, there would have been a further 58.7% increase in the fertility of those on ART. The factors in the decomposition analysis showed age (24.5%) WHO staging (17.2%) and contraception use (58.7%) was associated with lower fertility among those on ART while the BMI (11.0%) was correlated with higher fertility among those on ART, compared to those not on ART. Overall if these factors had been similar among the women on ART to those not on ART the increase would have been 93.8% higher, with an additional increase of 13.5 births per 1000 person years. In this case, the overall fertility rate would have been 68.45 per 1000 person years (Table 5).

**Table 4:** Fertility gaps of women on-ART and those not on-ART.

Exposure of interest	Number of Pregnancies	Person years/1000	Fertility rate/ 1000 years	95%C1 Lower	Upper
Post-ART	329	5.99	54.95	49.32	61.22
Pre-ART	194	4.79	40.52	35.20	46.64
Difference	130	1.20	14.43	14.12	14.58

**Table 5:** Decomposition analysis.

Characteristics	Total Fertility Rates difference		Difference in Endowments		Difference in coefficients	
	Value	%	Value	%	Value	%
**Overall**	**14.4**	**100.0**	**-13.5**	**-93.8**	**28.0**	**193.8**
**Characteristics**						
**Age (Total)**	**-3.4**	**-23.8**	**-3.5**	**-24.5**	**0.1**	**0.7**
40–49	0.8	5.8	-0.8	-5.9	1.7	11.7
35–39	-2.0	-14.1	-0.8	-5.2	-1.3	-8.8
30–34	-1.0	-6.7	0.0	-0.3	-0.9	-6.4
25–29	0.3	2.0	0.1	0.8	0.2	1.2
20–24	-0.6	-3.9	-0.6	-4.2	0.0	0.3
15–19	-1.0	-6.9	-1.4	-9.7	0.4	2.7
**Marital status (Total)**	**-1.6**	**-11.1**	**-0.2**	**-1.2**	**-1.4**	**-9.9**
Single	-0.6	-3.8	-0.1	-0.6	-0.5	-3.3
Divorced/Widowed	1.4	9.6	0.0	0.1	1.4	9.5
Married/Cohabiting	-2.4	-16.8	-0.1	-0.7	-2.3	-16.1
**CD4 cell count (Total)**	**-0.8**	**-5.8**	**-0.5**	**-3.2**	**-0.4**	**-2.6**
CD4 350 cells or less	0.4	2.9	-0.2	-1.6	0.6	4.4
CD4 Above 350 cells	-1.3	-8.7	-0.2	-1.6	-1.0	-7.1
**Contraceptive use (Total)**	**-21.6**	**-149.6**	**-8.5**	**-58.7**	**-13.1**	**-90.9**
Not using contraceptive	1.5	-160.3	-4.2	-29.4	5.8	-131.0
Using contraceptive	-23.1	10.7	-4.2	-29.4	-18.9	40.1
**BMI (Total)**	**-0.4**	**-2.7**	**1.6**	**11.0**	**-2.0**	**-13.8**
Below 18Kg/cm^2^	-1.7	-12.1	1.1	7.4	-2.8	-19.5
18-24Kg/cm^2^	0.9	6.4	0.4	2.9	0.5	3.5
Above 24Kg/cm^2^	0.4	3.0	0.1	0.8	0.3	2.2
**WHO Clinical staging (Total)**	**-1.5**	**-10.5**	**-2.5**	**-17.2**	**1.0**	**6.8**
WHO stage I	-1.4	-9.9	-1.6	-11.0	0.2	1.2
WHO stage II	-2.6	-17.9	0.2	1.5	-2.8	-19.4
WHO stage III	4.2	28.9	-0.1	-1.0	4.3	29.9
WHO stage IV	-1.7	-11.6	-1.0	-6.7	-0.7	-4.9

## Discussion

### Effect of ART on fertility rate

The findings from this study showed that the fertility rate among WLWH who were on ART was 50% higher than WLWH who had not yet started on ART. The higher fertility rate observed amongst women taking ART is consistent with other studies in Tanzania and across the region [8, 14, 15]. The use of ART has been reported in other studies to improve immunological functioning and increase female fecundity and fertility desire [18–20]. Therefore, availability of free ART in Tanzania in 2004 with ongoing training for healthcare providers may have elevated the confidence of WLWH and motivated them to resume sexual activity and consequently increased the chances of becoming pregnant [21].

### Factors associated with fertility rate change

The fertility rate of women in this study was inversely related with age and this findings aligns with previous studies [22–24]. These findings mirror the general population where women are mostly fertile between the ages of 20 and 24 years [24]. The younger women in this study were more likely to be pregnant compared to older women regardless of their ART status.

Modern family planning was inversely associated with fertility rate. This study reported lower use of modern family compared to general population (28% compared to 32%) [5]. In Tanzania, the use of any method of family planning among married women aged 15–49 is 32% [5]. WLWH have critical family planning needs [25]; studies in sub-Saharan Africa suggest that more than half of pregnancies in WLWH are unintended [26, 27]. Family planning needs of WLWH must be met to not only reduce mother-to-child HIV transmission but also as an essential component of HIV Prevention program. WLWH aspire to have children, and guidelines on safer conception are available in developed countries and in South Africa [28] to support their need to conceive while reducing horizontal or vertical HIV transmission. This becomes even more pertinent in the era of ART and in the context of the increasing fertility rates of HIV infected women. It is a call for other countries including Tanzania to consider including safer conception strategies into policy and reproductive child health guideline.

The higher fertility rate reported in 2014 was partly contributed by improved data capture system observed in the year ending 2013 and beginning of 2014. In one-third of all facilities used in this study, shifted from using paper-based, and moved to electronic-based data capture (CTC2 database). The increase of number of facilities using CTC2 may have contributed to higher new pregnancies reported into the CTC2 database. Given the same trend, if all facilities were using CTC2 database higher fertility rate may have been reported.

### Decomposition of the changes in uptake of HIV testing

This shows that, fertility increased by 14.43 births per 1000 person years in women on ART, compared to those not on ART. Other studies have reported higher fertility among WLWH using ART compared to those not on ART [8, 14, 15]. The use of contraception in this study increased after WLWH started using ART (from 23.4% to 34.2%). The overall fertility rate would have been at 66.5 per person years in women using ART, if the participants’ characteristics’ had remained the same as the women who were not using ART. In Tanzania, the national guideline for management of HIV and AIDS allows every person living with HIV to start ART immediately after HIV diagnosis. Therefore, as ART becomes more widely available for long time, the fertility rate among WLWH will increase and the gap between HIV positive women and general population will be reduced. A previous study has also reported a decrease over time in fertility gap between WLWH and those living without HIV [8]. Therefore, those models that assume lower fertility among WLWH when calculating predictions for national HIV prevalence need to consider the effect of ART among WLWH especially when more than 90% of WLWH who know their HIV status are currently on ART [2].

This study is based on recorded data in patients’ database the interpretation of the findings should consider the following limitations. First, the use of self-report for pregnancy that likely led to an underestimate of the true fertility rate because some of pregnancies resulting in spontaneous abortion prior to detection and some of the elective abortions may have been under reported however, this ascertainment challenge is not associated with use of ART.

## Conclusion

The fertility rate of WLWH on ART is higher than those pre-ART; although still lower than the fertility among women living without HIV. As ART continues to be widely available, and accessible we would expect its effect to impact the fertility trend in the general population. Therefore, there is a need to revisit the assumption that being an HIV positive women leads to a lower fertility rate when national HIV prevalence estimates are calculated. Additionally, the results presented in this research support the call to strengthen the integration of reproductive health counseling such as family planning in HIV services in order to prevent unwanted pregnancies and, for planned pregnancies, prevent HIV transmission to children and uninfected partners.

Differences in fertility rate between WLWH receiving ART and those not on ART exist. With more WLWH receiving ART at the point of HIV positive diagnosis, the gap in fertility between WLWH and those living without HIV will narrow. Therefore, the modelling of HIV trends in Tanzania using surveillance data from ANC after adjusting for the reduction in fertility of WLWH needs to be adjusted to allow for the impact of treatment.
